# pCADD: SNV prioritisation in *Sus scrofa*

**DOI:** 10.1186/s12711-020-0528-9

**Published:** 2020-02-07

**Authors:** Christian Groß, Martijn Derks, Hendrik-Jan Megens, Mirte Bosse, Martien A. M. Groenen, Marcel Reinders, Dick de Ridder

**Affiliations:** 1 Delft Bioinformatics Lab, University of Technology Delft, 2600GA Delft, The Netherlands; 2 Bioinformatics Group, Wageningen University & Research, 6708 PB Wageningen, The Netherlands; 3 Animal Breeding and Genomics, Wageningen University & Research, Wageningen, The Netherlands

## Abstract

**Background:**

In animal breeding, identification of causative genetic variants is of major importance and high economical value. Usually, the number of candidate variants exceeds the number of variants that can be validated. One way of prioritizing probable candidates is by evaluating their potential to have a deleterious effect, e.g. by predicting their consequence. Due to experimental difficulties to evaluate variants that do not cause an amino-acid substitution, other prioritization methods are needed. For human genomes, the prediction of deleterious genomic variants has taken a step forward with the introduction of the combined annotation dependent depletion (CADD) method. In theory, this approach can be applied to any species. Here, we present pCADD (p for pig), a model to score single nucleotide variants (SNVs) in pig genomes.

**Results:**

To evaluate whether pCADD captures sites with biological meaning, we used transcripts from miRNAs and introns, sequences from genes that are specific for a particular tissue, and the different sites of codons, to test how well pCADD scores differentiate between functional and non-functional elements. Furthermore, we conducted an assessment of examples of non-coding and coding SNVs, which are causal for changes in phenotypes. Our results show that pCADD scores discriminate between functional and non-functional sequences and prioritize functional SNVs, and that pCADD is able to score the different positions in a codon relative to their redundancy. Taken together, these results indicate that based on pCADD scores, regions with biological relevance can be identified and distinguished according to their rate of adaptation.

**Conclusions:**

We present the ability of pCADD to prioritize SNVs in the pig genome with respect to their putative deleteriousness, in accordance to the biological significance of the region in which they are located. We created scores for all possible SNVs, coding and non-coding, for all autosomes and the X chromosome of the pig reference sequence Sscrofa11.1, proposing a toolbox to prioritize variants and evaluate sequences to highlight new sites of interest to explain biological functions that are relevant to animal breeding.

## Background

Since humans started breeding animals, a key challenge has been to control the inheritance of traits. In farm animals, genetic gain has been achieved using pedigree information and statistical models. Since the introduction of genomic selection (GS) [1], breeding is transitioning from selecting animals based on visual inspection and pedigree data to approaches that exploit genetic information. However, given the complexity of genomes and the generally low level of knowledge about the relation between genotype and phenotype, undesirable alleles may accumulate, through genetic hitchhiking or genetic drift [2, 3] because of the small effective population size in livestock breeds under artificial selection.

Recent approaches incorporate whole-genome sequence data to improve genetic predictions. Because the number of tested single nucleotide variants (SNVs) is larger in whole-genome sequence data compared to array-based assays, truly causal genetic variants are more likely to be identified. While the use of whole-genome sequence data has improved genetic prediction, the improvements fall short of expectation and yield only moderate performance increases [4, 5], partly due to the inclusion of noise. Therefore, current strategies involve pre-weighting of potential candidate SNVs that have a higher probability of being causal. Several methods have been developed to score variants according to their putative deleteriousness and identify those that may have a detrimental effect on the fitness of individuals. Well-known variant prioritization tools include SIFT [6], PolyPhen2 [7], SNAP2 [8] and Provean [9]. However, these are limited to scoring (non-synonymous) variants in coding regions. In contrast, the combined annotation dependent depletion (CADD) [10] model that was developed to investigate SNVs in human populations, can score variants at any location in the genome. CADD is comparable to methods such as fitCons [11] and Linsight [12]: it captures signals of evolutionary selection across many generations and combines this with annotations—genomic features, epigenetic data, other predictors etc.—to estimate a deleteriousness score for a given variant. While CADD and similar models are well established and used to predict the effects of variants in the human genome [13–18], to date, they have not been applied to non-human species. In recent work [19], we applied CADD to mouse, and studied the effect of having a limited number of annotations, which is expected for non-model species, compared to the human case. The results demonstrated that applying the CADD methodology to non-human species is valid and powerful.

Here, we introduce pCADD (p for pig), a model based on the CADD methodology to create scores for the prioritisation of SNVs with respect to their putative deleteriousness in the genomes of wild and domesticated pigs (*Sus scrofa*). The aim of this paper is to assess the ability of pCADD to prioritize individual SNVs and genomic regions relative to their biological function. The ability of pCADD to score any SNV in the entire pig genome with respect to its predicted deleteriousness helps researchers and breeders to evaluate (newly) observed SNVs and rank potentially harmful SNVs that are propagated by breeding.

## Methods

**Fig. 1:**
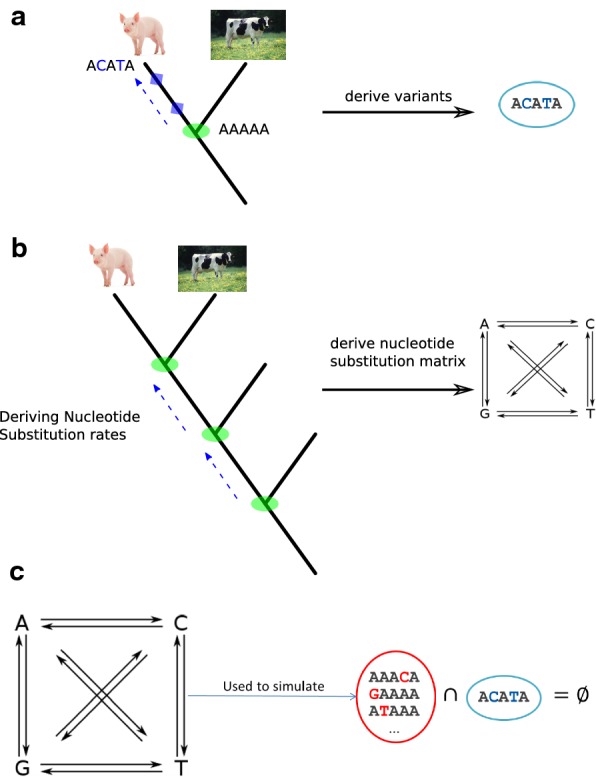
**a** Fixed alleles that differ between the investigated pig population and an inferred ancestor sequence are used as proxy benign/neutral SNVs. **b** First step of the simulation: differences between evolutionary differently distant ancestor sequences are identified and substitution rates are derived. **c** Simulation, second step: the derived substitution rates are used to simulate de novo variants that have not experienced any selection and thus are not depleted in deleterious variants

Briefly, the CADD model, which is a logistic regressor, assigns a deleteriousness score to a SNV based on a set of 867 genomic annotations such as DNA secondary structure, conservation scores, protein function scores and many more (see Additional file 1 and Additional file 2: Table S1). Model parameters are fitted based on a large training set, containing two classes of SNVs: derived (proxy benign/neutral) and simulated (proxy deleterious) SNVs. The set of derived SNVs is generated by identifying (nearly) fixed alleles in the species of interest that differ from those of a reconstructed ancestral genome (Fig. 1a). Proxy deleterious SNVs are simulated de novo mutations, which have not experienced any selection, thus deleterious variants are not depleted in this set (Fig. 1b, c).

With the pCADD model, every position in the pig genome can be scored with respect to its predicted deleteriousness. To differentiate more easily those SNVs that are potentially of interest, we created a PHRED-like score, which is similar to that in the original CADD approach [10]. To this end, the outcomes of the logistic regressor for all variants are ordered and transformed. The pCADD score is a log-rank score that ranges from ~ 95 to 0, with higher scores indicating more deleterious variants. The top 1% and 0.1% highest scored SNVs have a pCADD score higher than 20 and 30, respectively, thus the most deleterious variants are differentiated from the likely neutral ones. In the following, we describe the data used to train the pCADD model and demonstrate its use by performing several analyses.

### Training and test set construction

To create the set of derived variants, which consists of putatively benign/neutral variants, we identified (nearly) fixed alleles in a pig population that differ from those of the reconstructed ancestral genome of pig, cow and sheep (Fig. 1a, *Sus scrofa* [20], *Bos taurus* [21], *Ovis aries* [22]). These alleles have become fixed in the pig population due to genetic drift or positive selection, thus they are depleted in deleterious variants and can be assumed to have a benign or neutral effect. The ancestral sequence was obtained from the 25-eutherian-mammals EPO (Enredo, Pecan, Ortheus) [23, 24] multiple alignment files (MAF), downloaded from the Ensembl v.91 database. To avoid errors due to misaligned InDels, only SNVs that are not adjacent to another variant site, between the pig population and the inferred ancestor, were retained. The pig population used in our study included 384 individuals, representing 36 breeds, e.g. Asian and European, wild, commercial and local breeds (see Additional file 2: Table S2). For each site in the inferred ancestor, we selected an allele when its frequency was higher than 0.9 in the pig population and when it differed from the ancestral allele. Because the population includes pigs from many breeds, the number of functional variants that may have reached fixation due to founder effects in individual populations is limited. In addition, we removed sites that carry an allele at a frequency higher than 0.05 in the population and for which the alternate allele is equal to the ancestral allele. To simulate variants for the proxy deleterious set, substitution rates were derived from observed differences between more distant ancestors of pig (Fig. 1b, c). In particular, rates for nucleotide substitutions and CpG sites in window sizes of 100 kb were computed based on the inferred substitutions between the ancestral sequences of pig-cow, pig-horse and pig-dog. Only SNVs that were located at a site with a known ancestral allele of the pig-cow-sheep ancestor were simulated. These SNVs are de novo mutations that have a larger than uniform chance, with respect to other de novo mutations, to occur in the populations. Although these variations may have never occurred by chance along the evolutionary branch of pig, they may have also been actively selected against. In other words, these random mutations have a greater chance of being deleterious than benign [25], therefore the set of simulated variants is expected to be enriched in deleterious variants in comparison to the derived proxy benign/neutral set.

In total, 61,587,075 proxy benign/neutral SNVs were derived and a similar number of SNVs was simulated. To form the training and test sets, the dataset was randomly split into two sets with an equal number of samples from both classes. The training dataset contained 111,976,500 SNVs whereas the test set consisted of 11,197,650 SNVs. To assess the dependency on the genomic location of the variants, the test set was split into six overlapping subsets: (i) intergenic (non-cDNA) variants; (ii) all transcribed sites (cDNA); (iii) transcribed but not translated sites (5′UTR5, 3′UTR3 and introns); (iv) coding regions; (v) synonymous SNVs in coding regions and (vi) non-synonymous SNVs in coding regions.

### Variant annotation

Genomic annotations were obtained from the Ensembl Variant Effect Predictor (VEP v91.3) database [26] and supplemented by PhyloP [27], PhastCons [28] and GERP [29] conservation scores as well as Grantham [30] amino-acid substitution scores and predictions of secondary DNA structure (DNAshape) [31].

VEP-predicted consequences of SNVs were summarised in 14 categories. They were either used directly or combined with other data to create composite annotations (see Additional file 1 and Additional file 2: Table S3). Annotations that rely on a gene build, such as the SIFT protein score, reference and alternative amino-acid, variant position within a transcript and coding region were also used.

PhyloP and PhastCons scores are based on three differently sized multiple species alignments: a 6-taxa laurasiatheria, a 25-taxa eutherian-mammals and a 100-taxa vertebrate alignment. The laurasiatheria and eutherian-mammals alignments were downloaded from Ensembl [32] v91 whereas the 100-taxa vertebrate alignment was downloaded from UCSC [33, 34] (December 29, 2017). Next, PhyloFit [35] phylogenetic models were created for the laurasiatheria and eutherian-mammals alignments to compute PhastCons and PhyloP scores for pig. PhyloFit models for the 100-taxa vertebrate alignment were downloaded from the UCSC genome browser and used to compute PhastCons and PhyloP scores. PhastCons and PhyloP scores based on the 6- and 25-taxa alignments were directly computed for pig, while the scores for the 100-taxa alignment had to be first computed for the human reference GRCh38 and then mapped to Sscrofa11.1 using CrossMap [36]. To avoid a positive bias in predictive power in favour of PhastCons and PhyloP scores, the pig sequence was excluded from the generation of both sets of scores. Genomic evolutionary rate profiling (GERP) neutral evolution, GERP conservation, GERP constrained element and GERP constrained element p-values were retrieved from Ensembl91 using a custom Perl script.

Predicted differences in the secondary DNA structure between reference and alternative alleles were added as annotations to the dataset, as computed by DNAshape [31]: minor gap width (MGW), Roll, propeller twist (ProT) and helix twist (HelT).

After computing all annotation combinations, imputing missing values and recoding all categorical values to binary variables (see Additional file 1), the final number of features was equal to 867. Each feature was scaled by its standard deviation obtained from the variants in the training set.

### Construction of the model

We assigned class label 0 to the proxy benign/neutral variants and 1 to the proxy deleterious variants. Then, we trained a logistic regression classifier to predict the posterior probability of a variant being proxy deleterious. We used the logistic regression module provided by Graphlab v2.1 [37]. Based on previous experience and given the lack of a sufficiently large validation set, we applied the set of hyper parameters that were found to be optimal for mouse CADD19, i.e. L2-penalization was set to 0.1 and the number of iterations to 100. Feature rescaling, performed by the logistic regression function by default, was deactivated.

### Score creation

The pCADD scores were computed for all potential SNVs (3 per position) on the 18 autosomes and the X allosome. Each SNV was annotated with 867 genomic annotations and scored by the trained logistic regression model. Subsequently, these scores were sorted in descending order and assigned a pCADD score defined as $- 10*\log_{10} \left( {i/N} \right)$, with $i$ being the rank of a particular SNV and $N$ the total number of substitutions ($N$ = 7,158,434,598).

### Analyses

#### Codon analysis

From the Ensembl v.93 pig gene build, we retrieved 10,942 genes with only one annotated transcript to avoid complications due to overlapping transcripts. We created three sets, consisting of the minimum pCADD score found at a site, per transcript, one for each of the three positions of a codon. We computed one-tailed Mann–Whitney U-tests between each of the three sets. The resulting p-values were Bonferroni corrected. All calculations were performed in Python version 3 using SciPy v.1.1.0 [38] and Statsmodels v.0.9.0 [39].

#### miRNA analysis

We obtained all annotated (pre-)miRNA sequences from the Ensembl v93 database, i.e. 484 sequences, and, after removal of sequences that overlapped with any of the training SNVs, 294 sequences remained. As a second set, equally long sequences up- and downstream of the miRNA sequence were selected. For each position in both sets, the miRNA sequences and surrounding sequences were annotated with the maximum pCADD score. To test whether miRNA sequences had a significantly higher pCADD score than their neighbouring sequences, we applied a one-tailed Mann–Whitney U-test using SciPy v.1.1.0 in Python 3.

#### Intron analysis

We used the REST API of Ensembl v93 to download the intron coordinates of all 40,092 transcripts. We annotated all the sites in all the introns with the maximum pCADD score found at these sites. For each intron, we performed one-tailed Mann–Whitney U-tests to check if the investigated intron had a significantly higher pCADD score than all the other introns in the same transcript. p-values were Bonferroni corrected over all transcripts, per intron. To display the results, we normalized the number of rejected null-hypotheses by the number of conducted tests, which decreases as the number of introns increases.

#### Tissue analysis

We downloaded porcine Affymetrix expression data of several tissues published by Freeman et al. [40]. We selected the genes that were clustered and associated with a particular tissue in [38] and had a robust multi-array average (RMA) [41] expression level of at least 100 or more to filter out genes with no activity. Of these genes, we considered all the coding DNA sequences (CDS); if a particular CDS was present in more than one transcript, it was selected only once. In addition to the housekeeping genes, genes specific for 16 tissues were selected (cartilage-tendon, blood, cerebellum, dermal, epithelium, eye, kidney, liver, lung, muscle, neurone, pancreas, placenta, salivary gland, testis, and vasculature). All CDS were annotated with the maximum pCADD score found at each site of the CDS and merged into one set per tissue. Tissue sets were tested for higher scores than those of the housekeeping set with one-tailed Mann–Whitney U-tests; p-values were Bonferroni corrected. All calculations were done in Python 3 using the SciPy v.1.1.0 and Statsmodels v.0.9.0. modules.

## Results

In this study, we trained a CADD-like model for SNV prioritisation in the pig genome, which is referred to as pCADD. It is a linear regressor that is trained to differentiate between two classes of variants, a set of simulated variants, which is relatively more enriched in potentially deleterious variants than a set of derived variants, which is depleted in deleterious variants. The pCADD generated a score for every possible SNV of the Sscrofa11.1 reference genome on all autosomes and the X allosome. Then, these scores were tested on a held-out test set, they were used to evaluate seven SNVs with known functional effect and we examined whether they could discriminate between functional and non-functional sequences.

### pCADD data characteristics

The class distribution in the training and test sets were balanced, but subsets of SNVs found in different genomic regions displayed varying proportions of simulated and derived SNVs (Table 1). These imbalances were similar to those found for the human (hCADD) and mouse (mCADD) datasets in our previous study [19]. The largest difference among the three models is the total number of SNVs used for model training: ~ 31 million for hCADD, ~ 67 million for mCADD and ~ 112 million for pCADD. This results from the use of a more distant ancestor of the pig than the ancestors used for mouse in mCADD (mouse and rat) and for humans in hCADD (human and chimpanzee). A more distant ancestor yields more differences between the inferred ancestor and the species of interest, resulting in a larger derived class and, thus, in a larger total number of SNVs to create a balanced dataset.

**Table 1:** Number of SNVs and the relative proportions of the six subsets of the test set for pCADD

Pig partition	Number SNVs (proportion of test set)	Number of simulated SNVs	Number of derived SNVs	Class distribution (simulated/derived)
Test set	11,197,628 (100.00%)	5,598,814	5,598,814	50.00%/50.00%
Not cDNA	10,884,147 (97.20%)	5,404,059	5,480,088	49.65%/50.35%
cDNA	313,481 (2.80%)	194,755	118,726	62.13%/37.87%
Not CDS	154,622 (1.38%)	84,730	69,892	54.80%/5.20%
CDS	158,859 (1.42%)	110,025	48,834	69.26%/30.74%
Synonymous	75,216 (0.67%)	40,147	35,069	53.38%/46.62%
Missense	83,643 (0.75%)	69,878	13,765	83.54%/16.46%

### Increased discriminative power of pCADD with increased biological relevance of the sequence in which the queried SNVs are located

**Fig. 2:**
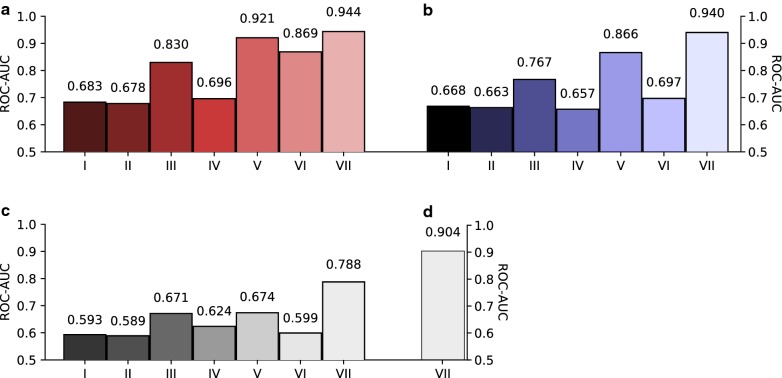
Prediction performances of different prioritization tools on test sets, representing various regions of the genome for which the number of features varies. I: whole test set; II: intergenic SNVs; III: transcribed SNVs; IV: SNVs in intron, 5′ and 3′ UTR; V: coding SNVs; VI: SNVs causing synonymous mutations; VII: SNVs causing missense mutations. **a** pCADD performance measured in ROC-AUC on the different subsets of the pig held-out test set. **b** mCADD test performance measured in ROC-AUC on the same genomic subsets in the mouse genome. **c** Performance of 6-taxa laurasiatheria PhastCons conservation score in the pig test set. **d** SIFT performance on missense causal SNVs in the pig test set

The performance of pCADD is evaluated by computing the receiver-operator-area under the curve characteristic (ROC-AUC) on a test set, which consisted of simulated and derived SNVs, none of which were used for training. The overall ROC-AUC on the entire test set is ~ 0.683, but differs considerably for six subsets of SNVs (Fig. 2a). The test sets are subsets of each other, with decreasing numbers of SNVs beginning with the whole test set and ending with the missense mutations. In transcribed regions of the genome, the scores are more discriminative than in non-transcribed regions, while in coding regions they are more discriminative than in non-coding regions such as the 5′UTR, 3′UTR and introns. The scores are most discriminative for missense mutations, which have the largest number of genomic annotations, resulting in high discriminative performance of the pCADD model.

These observations are in strong accordance with the earlier reported observations for the mCADD model for mouse (reproduced in Fig. 2b) [19], which was proven useful to identify truly deleterious mutations found in the Mutagenetix [42] data base, lifted from ClinVar [43] and others [19]. For all investigated SNV subsets, PhastCons [28] conservation scores based on the Ensembl 6-taxa laurasiatheria [32] displayed the same pattern across all subsets, but performed worse than pCADD (Fig. 2c). We used 6-taxa laurasiatheria PhastCons scores because, overall, they performed best on different subsets of the held-out test set (see Additional file 3: Figure S1). A similar difference in performance was observed when the performance of pCADD on missense mutations was compared to that of SIFT (Fig. 2d), which indicates the added value of pCADD over conventional approaches of identifying potential candidates.

### Selecting candidate SNVs based on their total score and on their relative rank in the surrounding region is meaningful

When we assessed examples of known causal SNVs (Table 2), they were enriched in the upper percentile of pCADD scores and were likely to be picked up as potential. The exception is 3:43952776T>G, one of two variants located in close proximity to a splice-site. In particular, it is located in an intron sequence, 4 bp upstream of an annotated splice site. Variants, which are located 1- and 2-bp upstream of the splice site have pCADD scores that range from 20.90 to 21.93, whereas the remaining variants in the same intron sequence have on average a pCADD score of ~ 2.96. Only 13 (out of 3450) other potential SNVs in that intron have a higher pCADD score. This puts the 3:43952776T>G SNV into the 99.6th percentile of the intron sequence in which it is located. None of the 13 potentially higher scored variants were observed in our population of 384 pigs, which makes 3:43952776T>G the highest scored SNV in that region.

**Table 2:** Seven well-known examples of causal SNVs with different effects on phenotype and their pCADD scores

Genomic location	Ref	Alt	pCADD	Percentile	Gene	Effect	Citations
6:146829589	G	A	22.868	99.5	*LEPR*	Missense: affects productive, fatness and meat quality traits in different genetic backgrounds	[44]
1:265347265	A	G	17.198	98.1	*NR6A1*	Missense: affects number vertebrae	[45]
17:57932233	A	C	23.322	99.5	*PCK1*	missense: causal mutation associated to intramuscular fat content, backfat thickness and meat quality in pigs	[46]
7:31281804	G	A	21.589	99.3	*PPARD*	Missense: affects ear size, fat metabolism, skin and cartilage development	[47]
12:38922102	G	A	21.848	99.3	*TADA2A*	Splice-donor: lethal recessives	[48]
3:43952776	T	G	10.144	90.3	*POLR1B*	Splice-region: lethal recessives	[48]
6:54880241	T	C	28.767	99.9	*PNKP*	Missense: lethal recessives	[48]

Both the pCADD scores and percentiles indicate their rank as candidate causal SNVs among all potential SNVs in the pig genome

### The third position of a codon is scored lower than the first two

To assess further if the model assigns different scores to sites with differing biological importance genome-wide, we tested whether the three positions in a codon are scored differently. Based on the fraction of non-synonymous mutations for each codon position, the second position should receive the highest score, followed by the first and third positions (see Additional file 3: Figure S2). To test this, we examined codons of genes that have only one known transcript, to avoid interference, which is expected by overlapping transcripts.

The table displays the counts of significant p-values between the three different positions in a codon. The columns indicate the positions that are tested to have higher pCADD scores than the positions in the rows. The numbers indicate how often the null hypothesis was rejected in 10,942 conducted tests.

Table 3 shows the number of significant tests when comparing the pCADD scores between two codon positions, across a gene, with each other (Bonferroni corrected, one-tailed Mann–Whitney U-tests). Among the 10,942 genes that were selected for this test, we found that the second codon position has a significantly higher pCADD score than the third for 8901 genes, and that the first codon position has a significantly higher pCADD score than the third for 8830 genes. Only for 3066 genes, did the second codon position score significantly higher than the first, while for 766 genes it was the opposite. Taken together, these results agree with our expectation, and indicate that pCADD scores do reflect deleteriousness. This was further confirmed by comparing the effect sizes, measured as ROC-AUC of the pairwise comparisons of codon positions (see Additional file 3: Figure S3).

**Table 3:** Number of significant Bonferroni corrected one-tailed Mann–Whitney U tests for pCADD scores compared at different codon positions

Smaller/larger	First	Second	Third
First	NA	3066	189
Second	766	NA	340
Third	8830	8901	NA

### miRNA regions are scored differently from those of neighbouring regions

**Fig. 3:**
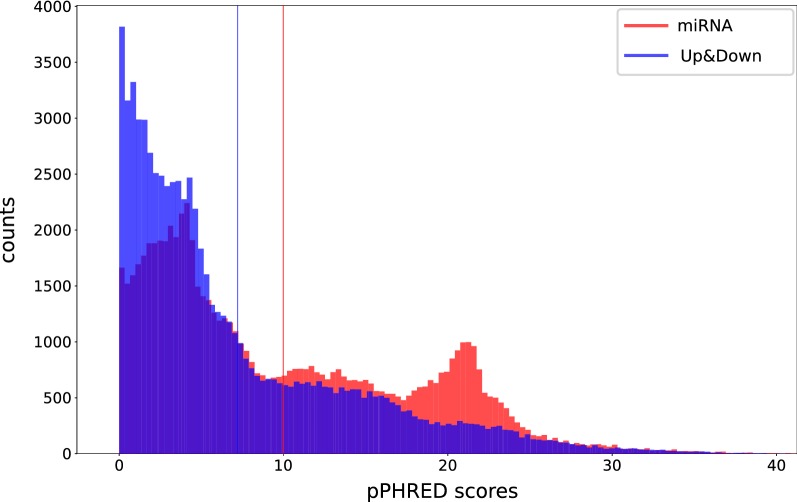
Histogram of the distribution of pCADD score for (pre-)miRNA transcripts and their surrounding up- and downstream regions. Vertical lines indicate the mean values of each distribution with a mean of 9.987 for miRNA and 7.205 for Up&Down. The one-tailed Mann–Whitney U-test between both distributions provided a p-value of 0.0 and a ROC-AUC of 0.613 in favour of miRNA over the Up&Down stream regions

We investigated whether pCADD scores are higher for functional non-coding sequences than for non-functional sequences up- and downstream. Variants in annotated (pre-)miRNA regions have significantly higher pCADD scores (p-value = 0.0, one-tailed Mann–Whitney U test; ROC-AUC = 0.613) than sites in up- and downstream regions (average pCADD scores of ~ 10 vs. ~ 7.2) (Fig. 3). This difference is largely due to an abundance of (pre-)miRNAs with pCADD scores around ~ 21 and a relatively smaller number of variants with a low score. For 164 miRNAs (~ 56%), the pCADD scores were significantly higher than those of the neighbouring regions (Bonferroni corrected, one-tailed Mann–Whitney U test).

### Among the introns of a transcript, the first one has the highest score

**Fig. 4:**
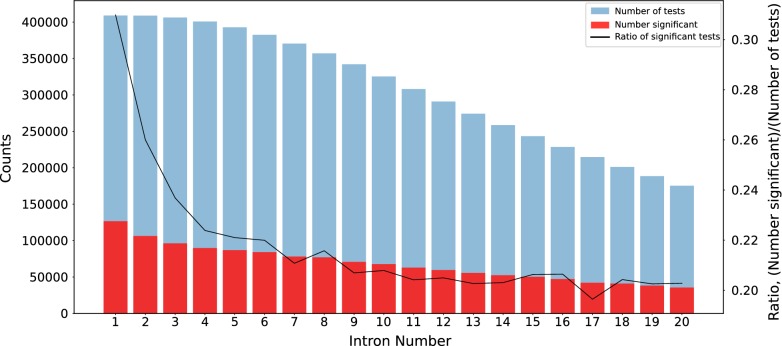
pCADD scores per intron compared to all other introns, for the first 20 introns. The blue bar indicates the number of introns tested against the intron of interest, the red bar shows how many of these tests resulted in an adjusted p-value < 0.05 (scale on the left axis). As the intron position increases, the number of tests that can be conducted decreases (with the number of transcripts that have at least that many introns). The black line represents the normalised number of significantly enriched introns, normalized by the number of conducted tests per intron position (scale on the right axis)

Chorev et al. [49] showed that regulatory elements are enriched in the first few introns of a transcript and that their number decreases with increasing intron position. Consequently, we expected to see decreasing pCADD scores with increasing intron position. To test this, we annotated every position in the intron region with the highest pCADD score for that position and calculated how often the scores in a particular intron are significantly higher than those across all other introns in the same transcript (Bonferroni corrected one-tailed Mann–Whitney U test). The results clearly show that introns closer to the transcription start site of a gene have higher pCADD scores (Fig. 4), which provide evidence for their biological relevance.

### Among all tested tissues, pCADD scores for salivary glands and neuronal tissue specific genes are the lowest and highest, respectively

Next, we investigated whether genes considered to be housekeeping genes have different (higher) pCADD scores than genes specifically expressed in certain tissues. The underlying assumption is that a mutation in a gene expressed in all tissue types has a much broader potential deleterious effect. We compared pCADD and PhyloP scores of genes specific for 16 tissues and also compared them (Bonferroni corrected one-tailed Mann–Whitney U test; ROC-AUC) to scores of a set of genes considered as housekeeping genes, i.e. expressed approximately equally in all tissues [40]. Based on pCADD scores, housekeeping genes had significantly higher scores for 12 of the 16 tissues examined (Table 4). Genes in three brain-derived tissues—cerebellum, eye, neuronal tissue—and in muscle tissue (smooth and skeletal) have on average a higher pCADD score than housekeeping genes. A ROC-AUC of 0.5 is the expected performance if the pCADD scores are randomly assigned to the genes of each set. This means that the larger the absolute difference is from 0.5, the clearer is the signal supporting that one set is larger than the other. We compared all tissue gene sets to housekeeping genes, this means that when the ROC-AUC is smaller than 0.5, the pCADD scores of the tissue associated gene set are generally larger than those of the housekeeping one and vice versa. In all the comparisons, the total effect size was small and did not differ from 0.5 by more than 0.122 (dermal tissue). The four tissues that displayed higher pCADD scores than housekeeping genes have in common that their cells do not divide anymore once they are fully differentiated. Mutations in these tissues may have a larger effect than in tissues with a high rate of cell division due to the inability of the tissue to replace cells, which leads to scarring and eventually tissue failure. Thus, genes specific to these four tissues are more likely conserved than those specific to other tissues, resulting in overall higher pCADD scores. This is supported by the analysis with conservation scores (Table 4), which showed that these genes were more conserved than the housekeeping genes. Tissues such as dermal and salivary gland show the lowest pCADD scores and high rates of cell division. These tissues are likely more tolerant to germline mutations since they must adapt to changes in diet and climate, thus their tissue-specific genes have a higher variability, resulting in lower pCADD scores.

**Table 4:** Test results between tissue-specific gene sets and housekeeping genes

Tissue	pCADD p-value (tissue < housekeeping)	pCADD ROC-AUC (housekeeping vs. tissue)	PhyloP p-value (tissue < housekeeping)	PhyloP ROC-AUC (housekeeping vs. tissue)
All tissues	2 × 10^−1^	0.500	1	0.467
Blood	3 × 10^−122^	0.512	1	0.481
Cartilage-Tendon	3 × 10^−35^	0.511	1	0.453
Cerebellum	1	0.480	1	0.487
Dermal	0	0.622	0	0.681
Epithelium	0	0.538	1 × 10^−29^	0.515
Eye	1	0.475	1	0.456
Kidney	2 × 10^−100^	0.515	1	0.468
Liver	1 × 10^−54^	0.510	9 × 10^−1^	0.490
Lung	6 × 10^−8^	0.506	1 × 10^−2^	0.503
Muscle	1	0.491	1	0.468
Neuronal	1	0.443	1	0.400
Pancreas	1 × 10^−310^	0.558	3 × 10^−81^	0.559
Placenta	1 × 10^−145^	0.529	1	0.469
Salivary-gland	7 × 10^−48^	0.519	1	0.478
Testis	0	0.558	1	0.478
Vasculature	0	0.558	1	0.454

We tested if tissue-specific genes have significantly lower pCADD scores than housekeeping genes, using pCADD and PhyloP scores (25-taxa mammalian alignment). The ROC-AUC scores display the likelihood that a random sample from the scores of the housekeeping genes is greater than that from the scores of tissue-specific genes

### Differentiation between functional and non-functional sequences is greater with pCADD than conservation scores

Conservation scores are often used to evaluate the potential importance of sequences and to evaluate if a particular candidate SNV may have a deleterious effect. They are also useful to put our own results into perspective and assess conventional sequence prioritisation methods.

Similar to the section “miRNA regions are scored differently from those of neighbouring regions”, we annotated the pre-miRNAs and their associated up- and downstream regions with PhyloP conservation scores (based on 25-taxa mammalian alignment) and performed the same analysis by computing significance tests to check if miRNA sequences have higher pCADD scores than those in their neighbouring regions. We chose 25-taxa PhyloP scores because these have the largest coverage of the pig genome among all conservation scores used in this study (see Additional file 2: Table S4). The results are in Additional file 3: Figure S4 and are very similar to those from the analysis using pCADD scores, with an almost identical p-value close to 0 (1e−225) and a ROC-AUC value of 0.595, which indicates a slightly worse separation between both classes of sequences than when using pCADD.

Likewise, we evaluated the intron positions relative to each other using the same PhyloP conservation scores to annotate intron sequences. The results in Additional file 3: Figure S5 show a similar pattern of decreasing importance with increasing intron position as observed when the introns are annotated with pCADD scores. Major differences between the analysis using pCADD and conservation scores is that the total number of introns, which can be annotated with conservation scores is smaller, resulting in 81,743 fewer tests compared with pCADD. Furthermore, the ratio between the total number of tests and the number of tests with an adjusted significant p-value is smaller when conservation scores are used, which indicates that conservation scores are less discriminative between different intron positions.

We annotated tissue-specific and housekeeping genes with PhyloP conservation scores to investigate whether the differentiation between both sets of genic regions followed the same pattern. Twelve tissue-specific gene sets displayed significantly lower pCADD scores than housekeeping genes, whereas only four tissues had a significantly lower conservation score. The larger total differences in ROC-AUC scores obtained by using PhyloP scores compared to pCADD scores indicate that the variations between tissue gene sets are larger when using PhyloP.

The worse performance of PhyloP scores to distinguish between pre-miRNA and surrounding regions is supported by the lower ratio of significant tests in the intron analysis, which indicates that PhyloP scores have less specificity for functional elements than pCADD scores.

### Predicted intergenic SNVs with high pCADD scores are often associated with lncRNA and may indicate missing annotations

**Fig. 5:**
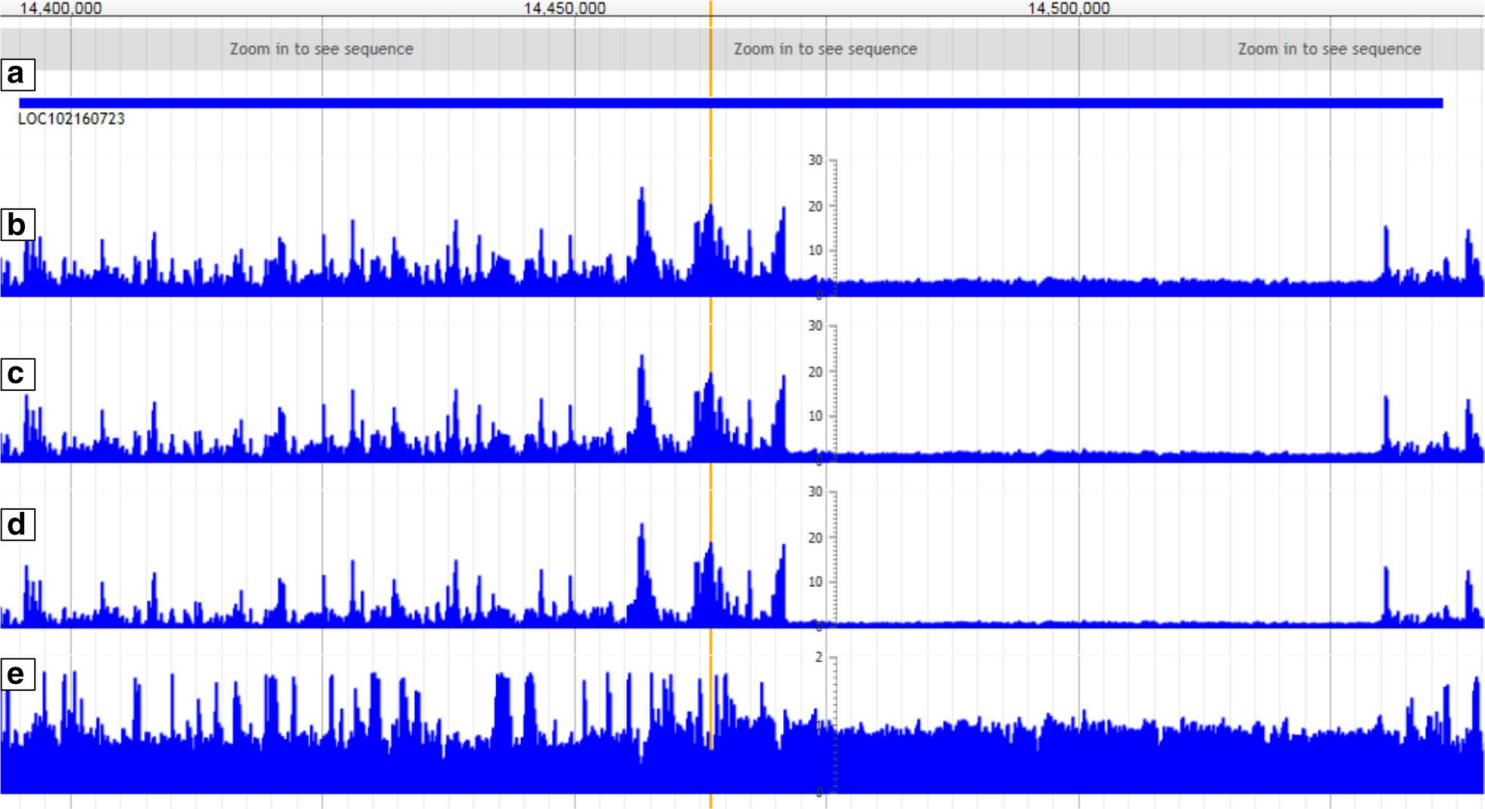
Visualization of the three potential nucleotide substitutions at each position in the genome, each with their own predicted pCADD score. To visualize pCADD scores in JBrowser, we created tracks for the maximum, median and minimum scores at each position. The fourth track displays the standard deviation among the three scores to identify more easily sites of variable deleteriousness. The yellow vertical bar is located at position 5:14463457, indicating the site of the top scoring SNV in Table 5. This SNV is considered intergenic according to the Ensembl gene build but located within a lncRNA according to the NCBI genebuild. **a** NCBI gene build track, showing the genomic region belonging to lncRNA LOC102160723. **b**–**d** the maximum, median and minimum pCADD scores for each position in the displayed region. **e** The standard deviation of pCADD scores at each position

To examine the utility of pCADD scores for the prioritization of SNVs, we investigated whether they can help in the identification of intergenic candidate SNVs that segregate between two closely related Large White pig breeding populations. We scored intergenic SNVs that were unique for either of these pig populations by multiplying their pCADD score with the allele frequency and selected the top 20 highest scored SNVs for each population. Since the pCADD model is based on the Ensembl pig annotations [50] (Ensembl gene annotation update e!90 Sscrofa11.1), we matched the selected 40 SNVs with NCBI’s pig gene build [51] to determine whether the model captures non-annotated genomic features. We found that 16 of the 40 SNVs are located within a (NCBI) coding region (one example shown in Fig. 5) and six SNVs overlap with a (NCBI) long non-coding RNA (Table 5).

**Table 5:** Top 40 SNVs according to pCADD^*^Alt:Frq that are presumably intergenic according to the Ensembl *Sus scrofa* gene build, annotated with NCBI

Chr	Pos	Ref:Frq	Alt:Frq	pCADD	pCADD*Alt:Frq	NCBI-gene build	Human-ensembl-gene build
5	14463457	T:0.014	C:0.986	26.559	26.185	lncRNA	
10	45490687	G:0.007	T:0.993	24.175	24.000	RSU1	
9	88698813	C:0.021	G:0.979	24.433	23.909		lncRNA
*6*	*149549021*	*T:0.007*	*C:0.993*	*23.714*	*23.544*		
18	30883512	G:0.045	A:0.955	24.211	23.111	lncRNA	
14	102653354	A:0.007	G:0.993	23.216	23.052	lncRNA	
3	35533299	C:0.029	T:0.971	23.729	23.041	RBFOX1	
8	16080284	T:0.021	G:0.979	23.540	23.035	KCNIP4	
8	16090742	A:0.007	C:0.993	23.188	23.0248	KCNIP4	
9	88631400	T:0.037	C:0.963	23.855	22.978		lncRNA
13	11996804	A:0.068	G:0.932	24.518	22.846		miscRNA
8	16069085	C:0.014	T:0.986	23.148	22.817	KCNIP4	
*1*	*270976051*	*G:0.057*	*A:0.943*	*24.148*	*22.768*		
*12*	*10080096*	*C:0.029*	*T:0.971*	*23.417*	*22.738*		
*15*	*134154371*	*G:0.028*	*A:0.972*	*23.388*	*22.729*		
*17*	*15317464*	*T:0.035*	*C:0.965*	*23.437*	*22.611*		
8	16126909	T:0.145	G:0.855	26.331	22.515	KCNIP4	
14	102708028	T:0.007	C:0.993	22.622	22.463		lncRNA
17	8460314	T:0.007	A:0.993	22.607	22.448		FAT1
3	2721065	C:0.016	T:0.984	22.794	22.438		SDK1
8	2274651	T:0.006	C:0.994	24.861	24.721	lncRNA	
14	41547002	T:0.006	C:0.994	24.651	24.511	MYO1H	
9	88656584	T:0.023	C:0.977	24.606	24.047		lncRNA
13	145274213	A:0.031	G:0.969	24.336	23.576	ZBTB20	
5	14463352	A:0.006	G:0.994	23.526	23.393	lncRNA	
*2*	*135162568*	*A:0.011*	*C:0.989*	*23.305*	*23.043*		
13	196634107	A:0.011	C:0.989	23.190	22.930		lncRNA
*13*	*203405436*	*G:0.006*	*A:0.994*	*23.046*	*22.917*		
*17*	*15317464*	*T:0.022*	*C:0.978*	*23.436*	*22.910*		
*13*	*203404345*	*T:0.017*	*G:0.983*	*23.239*	*22.842*		
18*	4227731	C:0.006	A:0.994	22.839	22.710		
*13*	*203405428*	*T:0.006*	*G:0.994*	*22.663*	*22.535*		
13	145279451	A:0.019	G:0.981	22.960	22.512	ZBTB20	
*15*	*134347171*	*T:0.006*	*G:0.994*	*22.633*	*22.506*		
5	25295998	A:0.011	G:0.989	22.731	22.476	lncRNA	
*15*	*134154371*	*G:0.040*	*A:0.960*	*23.387*	*22.457*		
*18*	*42017803*	*T:0.017*	*G:0.983*	*22.811*	*22.427*		
*15*	*134347189*	*G:0.006*	*C:0.994*	*22.471*	*22.345*		
8	16126909	T:0.152	G:0.848	26.331	22.337	KCNIP4	
14	138794865	A:0.006	G:0.994	22.411	22.285		lncRNA

When no NCBI gene annotation was found, SNVs were mapped to hg38 and the Human Ensembl gene build was used. Italics: SNVs that are intergenic in the three gene builds, yet found in regions with conserved synteny

*SNV located in a region unannotated in any gene build

In addition, we mapped the genomic locations of the candidate SNVs to the human assembly GRCh38.p12 and Ensembl gene builds, which revealed nine additional genic regions that consisted of six lncRNAs, one region considered as a miscRNA and two genes. For all 40 SNVs, synteny of the surrounding genes was conserved except for 18:4227731C>A. The relatively large number of prioritized SNVs that overlap with lncRNAs can be explained in two ways. First, there might be a considerable number of missing annotations in the gene builds that we used because the RNA-seq databases are incomplete and are the basis for lncRNA annotations. Second, although the lncRNA functions are conserved due to islands of strong conserved regions [52], the architecture of their sequences experience constant restructuring and weak sequence conservation across species [51, 52].

The highest scored SNVs (in terms of pCADD score multiplied by alternative allele frequency) for which no genic annotation was found (6:149549021T>C) (Table 5), is located in an island with high pCADD scores within a region that contains several of such small islands (see Additional file 3: Figure S6). This region starts with a highly H3K27Ac acetylated region, which indicates an enhancer site. Such a pattern is uncommon for intergenic regions and could indicate a missing annotation in the gene builds used in our study.

## Discussion

We used a method that provides scores for the prioritization of SNVs with respect to their putative deleteriousness, from which we derived functional relevance for the genomes of pig. The method is based on the creation of a set of derived variants from an inferred common ancestor sequence that can be assumed to be depleted in deleterious variants and a set of simulated variants that are likely to be enriched in variants with a deleterious effect. It is important to note that while it is reasonable to assume that the proxy benign/neutral are truly benign/neutral variants, the simulated putative deleterious variants may also encompass a relatively large proportion of actually neutral variants.

Founder effects in pig populations may lead to the accumulation of functional variants, with both benign and deleterious variants receiving a relatively high pCADD score. This means that pCADD scores are useful to prioritize SNVs of interest, but that assessing deleteriousness may need additional information or experiments. For example, the missense variant 1:265347265A>G (pCADD:21.848), which is responsible for an increased number of vertebrae and can be considered benign given current breeding goals, and the deleterious lethal recessive splice variant 12:38922102G>A, have similar pCADD scores (pCADD: 17.198) (Table 2).

We evaluated the generated pCADD scores on a held-out test set and reported performances on different genomic subsets, which we compared to results of our previous study on mouse. Due to the nature of the procedure, the test performance can only indicate if the training algorithm has picked up patterns of features that are predictive for the simulated variants and if the performance varies with the genomic region. It has to be emphasized that only performance trends can be meaningfully compared between the different mCADD/pCADD models due to the different datasets used for computation. In spite of the large number of neutral variants, which is expected in both sets of variants, the performance seems to indicate that patterns to differentiate between the derived and simulated datasets have been picked up and can be used to evaluate variants and regions based on their potential interest.

The performance of pCADD scores to discriminate between simulated and derived variants in the test set increased as the number of features increased, depending on the genomic regions in which they are embedded. The consequence is that missense mutations are the best classified, although the most interesting application of pCADD is to annotate non-coding and intergenic variants, for which a plethora of functional candidates exist but there are only a few methods for further prioritization. As shown for the splice-region variant 3:43952776T>G, the ranking of a variant relative to its neighbouring sequence in the same sequence category (introns, exons, intergenic, etc.) can provide information that helps to prioritize such variants.

Furthermore, we used PHRED-like scores to rate different sequences with known biological function. We compared the scores for the three positions in a codon and found that less redundant positions achieve higher pCADD scores. Moreover, regulatory sequences could be clearly distinguished from their neighbouring regions (i.e. high scores in miRNAs). In addition, our model supports the higher frequency of regulatory elements in the first few introns of a transcript, and thus has the potential of scoring not only individual SNVs but also of using a summary score per site to annotate entire regions to identify potential sub-regions of interest. This is a clear advantage compared to alternative methods to evaluate non-coding sequences, such as conservation scores, which may not be available for the entirety of the genome. This was the case in the analysis of intron sequences, for which more than 80,000 fewer tests could be conducted due to missing conservation scores. Using pCADD, candidate regions in which annotations are potentially missing can be identified. For example, no annotation was found for the 6:149549021T>C SNV, even though pCADD scores were within a range typical for exons and displayed patterns of islands of high importance (see Additional file 3: Figure S6), which is more compatible with coding regions than with intergenic regions. Ensembl gene annotations rely strongly on transcript data from public databases, which implies that incomplete databases may lead to missing gene annotations. This is especially the case for species that are less well studied than model organisms or humans. In addition, if the genes in question are not ubiquitously expressed, they can be absent from the data of the sequenced tissue. The same is true for genes, the expression of which depends on developmental-, disease- or physiological state, as is the case for many lncRNAs [53].

We compared genes specific for 16 different tissues against (presumed) housekeeping genes [40]. Our assumption was that the ubiquitously and generally more highly expressed housekeeping genes [54] should have globally higher scores than tissue-specific genes. Although the absolute effect size was small, significantly higher scores were attributed to genes specific to cerebellum, eye, neuronal and muscle tissue. Brain-derived tissues (cerebellum, eye, neuronal tissue), in particular, displayed the largest effect sizes. On the one hand, brain tissue has experienced major development changes during the time period between 535 and 310 Mya ago, i.e. increased expression and gain of functions of paralogs of brain-specific genes [54, 55]. Since then and during the entire mammalian development, the expression of paralogs of brain-specific genes is lower than that observed in other tissues [56], which indicates the fine balancing that acts to keep the brain functional. This emphasizes the extreme importance of brain-specific genes for survival and probably their low tolerance to mutations, compared to housekeeping genes. On the other hand, dermal tissue (epithelium) is one of the most ancient tissues in the evolution of metazoans and has highly conserved developmental pathways, which include genes that are involved in the adaptation to specific environmental changes and have overall lower pCADD scores than housekeeping genes.

Among the most important features for the pCADD model are conservation scores. They are annotated for large fractions of the genome (see Additional file 2: Table S4), and thus they heavily influence training. This is supported by our investigation of various tissues, which showed that particularly high scores were assigned to expected strongly conserved regions. Deleterious effects that are not captured by sequence conservation, such as changes in the epigenome or in relatively variable regions, are expected to have lower scores. This becomes problematic when the species of interest has experienced recent genetic bottlenecks and has been subjected to very strong selection, which change the species’ genotype, as is the case for domesticated species. In this case, the patterns observed from evolutionary changes may not be accurate to evaluate recent changes. However, not all the regions in the genome are subject to substitution, neither in natural nor in domesticated environments. There are exceptions to this rule, such as the reported missense mutations in Table 2, which are causal for a change in the number of vertebrae, ear size, meat quality and fat content, and have high scores, which support the use of pCADD for variant prioritization.

## Conclusions

The CADD approach is widely used in humans [13–18] and, based on our findings, it seems to be a suitable approach for pig (and other non-human species). Variants that distinguish populations can be ranked with respect to their pCADD score and allele frequency to find potential candidates for phenotypes expressed in the studied populations. pCADD could become a valuable tool in pig breeding and conservation. It can be used to score variants with a potential negative effect in small-sized endangered local pig breeds, but also help prioritize high-impact variants in genomic prediction to further enhance genomic selection.

## Supplementary information

**Additional file 1.** Annotation pre-processing. Description of the pre-processing procedure of the datasets used to train the pCADD model.

**Additional file 2: Table S1.** Overview of genomic annotations that build the basis for features used to train the pCADD model. Overview and short description of genomic annotations and their imputed values in the case of missing data. **Table S2.** Overview of the pig populations used in this study. List of pigs for which the high-frequency SNVs were added to the set of the putative benign (derived) variants to generate the training set. SNVs were called based on whole-genome sequence data. **Table S3.** VEP consequences summaries. VEP variant consequences, summarized into 14 categories. If multiple annotations exist for the same variant, the predicted variant consequence is selected according to the displayed hierarchy, starting at 1 and ending at 14. **Table S4.** Conservation score coverage of the pig genome. Coverage of the pig genome by the different conservation scores used in the pCADD model (see Table S1). Y-chromosome, mitochondrial and unplaced scaffolds were excluded in pCADD and the conservation score calculations.

**Additional file 3: Figure S1.** Prediction performances of six conservation scores on test sets, representing different regions of the genome for which different numbers of features are available. I: whole test set; II: Intergenic SNVs; III: transcribed SNVs; IV: SNVs in introns, 5′ and 3′ UTRs; V: coding SNVs; VI: SNVs causing synonymous mutations; VII SNVs causing missense mutations. **Figure S2.** Codon redundancy displayed in the JBrowser genome browser using pCADD scores. The third position in a codon is more redundant than either of the two other positions. This is reflected in the scores, here an example of the end of the second exon of the *MACC1* gene. *MACC1* is located on the reverse strand. **Figure S3.** Effect sizes measured as ROC-AUC of the pairwise comparisons of pCADD scores of the three codon sites for all transcripts. The pCADD scores for the third and second codon positions differ generally the most (mean of ~ 0.232), thus their effect sizes have the largest absolute distance to 0.5. A ROC-AUC of 0.5 would indicate that no set of scores is larger than the other. The score indicates that the third position has a generally lower pCADD scores than the second position. The effect sizes of pCADD scores between the third and first codon positions (mean ROC-AUC ~ 0.277) also indicate that the third position is generally evaluated to be less deleterious than the first. In contrast, effect sizes between the second and first codon position are on average larger than 0.5 (mean of ~ 0.554) with the second codon position having a generally higher pCADD score than the first, which confirms that the second codon position is the most consequential when mutated. The effect sizes between the third and second codon positions as well as the third and first codon positions are more dispersed than between the second and first codon positions, probably due to the relatively larger variance in impact of a change at the third position than at the other two positions. **Figure S4.** Histogram of conservation score distribution of (pre-)miRNA transcripts and their surrounding up- and downstream regions. Vertical lines indicate the mean values of each distribution with a mean of 0.382 for miRNA and 0.211 for Up&Down. The one-tailed Mann–Whitney U-test between both distributions provided a p-value of 1e-225 and a ROC-AUC of 59.54%. The conservation score used to annotate the transcripts and their surrounding regions are the 25-taxa-Mammalian PhyloP score shown in Additional file 2: Table S4. **Figure S5.** Comparison of the 25-taxa-Mammalian PhyloP scores per intron with all other introns, for the first 20 introns. The blue bar indicates the number of introns tested against the intron of interest, the red bar how many of these tests resulted in an adjusted p-value < 0.05 (scale on the left axis). As the intron position increases, the number of tests that can be conducted decreases (with the number of transcripts that have at least that many introns). In black, the normalised number of significantly enriched introns, normalized by the number of conducted tests per intron position (scale on the right axis). **Figure S6.** pCADD scores show a pattern of high scores in a presumably intergenic region. The yellow bar indicates the location of the SNV 6:149549021T > C. It is embedded in a presumably intergenic region without any gene annotations in the pig genebuild of Ensembl and NCBI and the Ensembl genebuild of human when mapped to the human genome. The region contains many islands of high pCADD scores, which are untypical for intergenic regions, and starts with an active enhancer region (peaks in H3K27Ac). The 5′region of the enhancer site displays patterns as expected for intergenic regions.

## Data Availability

pCADD scores, partitioned per chromosome, compressed via bgzip and tabix indexed for fast access, can be downloaded following this link (~ 5–1 GB): http://www.bioinformatics.nl/pCADD/indexed_pPHRED-scores/ To create tracks for genome browsers we provide the maximum, median, minimum, and standard deviation summaries of each site, partitioned per chromosome. All files are compressed with bgzip and tabix indexed and can be downloaded following this link (~ 1.7 GB to ~ 350mb): http://www.bioinformatics.nl/pCADD/indexed_pPHRED-summary-scores/ Scripts and data to recreate the figures in this article can be downloaded from the following link: https://git.wur.nl/gross016/pcadd-scripts-data

## References

[1] Meuwissen THE, Hayes BJ, Goddard ME (2001). Prediction of total genetic value using genome-wide dense marker maps. Genetics.

[2] Good BH, Desai MM (2014). Deleterious passengers in adapting populations. Genetics.

[3] Gillespie JH (2001). Is the population size of a species relevant to its evolution?. Evolution.

[4] Pérez-Enciso M, Rincón JC, Legarra A (2015). Sequence- vs. chip-assisted genomic selection: Accurate biological information is advised. Genet Sel Evol..

[5] Brøndum RF, Su G, Janss L, Sahana G, Guldbrandtsen B, Boichard D (2015). Quantitative trait loci markers derived from whole genome sequence data increases the reliability of genomic prediction. J Dairy Sci.

[6] Ng PC, Henikoff S (2001). Predicting deleterious amino acid substitutions. Genome Res.

[7] Adzhubei IA, Schmidt S, Peshkin L, Ramensky VE, Gerasimova A, Bork P (2010). A method and server for predicting damaging missense mutations. Nat Methods.

[8] Hecht M, Bromberg Y, Rost B (2015). Better prediction of functional effects for sequence variants. BMC Genomics.

[9] Choi Y, Chan AP (2015). PROVEAN web server: a tool to predict the functional effect of amino acid substitutions and indels. Bioinformatics.

[10] Rentzsch P, Witten D, Cooper GM, Shendure J, Kircher M (2019). CADD: predicting the deleteriousness of variants throughout the human genome. Nucleic Acids Res.

[11] Guiko B, Hubisz MJ, Gronau I, Siepel A (2015). Probabilities of fitness consequences for point mutations across the human genome. Nat Genet.

[12] Huang YF, Gulko B, Siepel A (2017). Fast, scalable prediction of deleterious noncoding variants from functional and population genomic data. Nat Genet.

[13] Lek M, Karczewski KJ, Minikel EV, Samocha KE, Banks E, Fennell T (2016). Analysis of protein-coding genetic variation in 60,706 humans. Nature.

[14] van der Velde JK, Kuiper J, Thompson BA, Plazzer JP, van Valkenhoef G, de Haan M (2015). Evaluation of CADD scores in curated mismatch repair gene variants yields a model for clinical validation and prioritization. Hum Mut..

[15] Balasubramanian S, Fu Y, Pawashe M, McGillivray P, Jin M, Liu J (2017). Using ALoFT to determine the impact of putative loss-of-function variants in protein-coding genes. Nat Commun..

[16] Banaganapalli B, Rashidi O, Saadah OI, Wang J, Khan IA, Al-Aama JY (2017). Comprehensive computational analysis of GWAS loci identifies CCR2 as a candidate gene for celiac disease pathogenesis. J Cell Biochem.

[17] Mesbah-Uddin M, Elango R, Banaganapalli B, Shaik NA, Al-Abbasi FA (2015). In-silico analysis of inflammatory bowel disease (IBD) GWAS loci to novel
connections. PLoS One.

[18] Al-Tassan NA, Whiffin N, Hosking FJ, Palles C, Farrington SM, Dobbins SE (2015). A new GWAS and meta-analysis with 1000Genomes imputation identifies novel risk variants for colorectal cancer. Sci Rep..

[19] Groß C, de Ridder D, Reinders M (2018). Predicting variant deleteriousness in non-human species: applying the CADD approach in mouse. BMC Bioinformatics.

[20] Groenen MAM, Archibald AL, Uenishi H, Tuggle CK, Takeuchi Y, Rothschild MF (2012). Analyses of pig genomes provide insight into porcine demography and evolution. Nature.

[21] Zimin AV, Delcher AL, Florea L, Kelley DR, Schatz MC, Puiu D (2009). A whole-genome assembly of the domestic cow, *Bos taurus*. Genome Biol..

[22] Jiang Y, Xie M, Chen W, Talbot R, Maddox JF, Faraut T (2014). The sheep genome illuminates biology of the rumen and lipid metabolism. Science.

[23] Paten B, Herrero J, Beal K, Fitzgerald S, Birney E (2008). Enredo and Pecan: genome-wide mammalian consistency-based multiple alignment with paralogs. Genome Res.

[24] Paten B, Herrero J, Fitzgerald S (2008). Genome-wide nucleotide-level mammalian ancestor reconstruction. Genome Res.

[25] Doniger SW, Kim HS, Swain D, Corcuera D, Williams M, Yang SP (2008). A catalog of neutral and deleterious polymorphism in yeast. PLoS Genet.

[26] McLaren W, Gil L, Hunt SE, Riat HS, Ritchie GR, Thormann A (2016). The ensembl variant effect predictor. Genome Biol.

[27] Pollard KS, Hubisz MJ, Rosenbloom KR, Siepel A (2010). Detection of nonneutral substitution rates on mammalian phylogenies. Genome Res.

[28] Siepel A, Bejerano G, Pedersen JS, Hinrichs AS, Hou M, Rosenbloom K (2005). Evolutionarily conserved elements in vertebrate, insect, worm, and yeast genomes. Genome Res.

[29] Davydov EV, Goode DL, Sirota M, Cooper GM, Sidow A, Batzoglou S (2010). Identifying a high fraction of the human genome to be under selective constraint using GERP++. PLoS Comput Biol.

[30] Grantham R (1974). Amino acid difference formula to help explain protein evolution. Science.

[31] Zhou T, Yang L, Lu Y, Dror I, Dantas Machado AC (2013). DNAshape: a method for the high-throughput prediction of DNA structural features on a genomic scale. Nucleic Acids Res.

[32] Hunt SE, McLaren W, Gil L, Thormann A, Schuilenburg H, Sheppard D (2018). Ensembl variation resources. Database..

[33] Kent WJ, Sugnet CW, Furey TS, Roskin KM, Pringle TH, Zahler AM (2002). The human genome browser at UCSC. Genome Res.

[34] Casper J, Zweig AS, Villarreal C, Tyner C, Speir ML, Rosenbloom KR (2018). The UCSC genome browser database : 2018 update. Nucleic Acids Res.

[35] Siepel A, Haussler D (2004). Phylogenetic estimation of context-dependent substitution Rates by maximum likelihood. Mol Biol Evol.

[36] Zhao H, Sun Z, Wang J, Huang H, Kocher J, Wang L (2014). CrossMap: a versatile tool for coordinate conversion between genome assemblies. Bioinformatics.

[37] Turi. Graphlab create. https://turi.com/index.html. Accessed 14 Mar 2017.

[38] Jones E, Oliphant T, Peterson P. Scipy: open source scientific tools for Python. http://www.scipy.org. Accessed 3 June 3 2019.

[39] Seabold S, Perktold J. Statsmodels : econometric and statistical modeling with Python. In: Proceedings of the 9th Python in Science Conference: 28 June–3 July 2010, Austin; 2010. p. 57–61. http://statsmodels.sourceforge.net/.

[40] Freeman TC, Alasdair I, Baillie JK, Beraldi D, Barnett MW, Dorward D (2012). A gene expression atlas of the domestic pig. BMC Biol.

[41] Irizarry RA, Hobbs B, Collin F, Beazer-Barclay YD, Antonellis KJ, Scherf U (2003). Exploration, normalization, and summaries of high density oligonucleotide array probe level data. Biostatistics..

[42] Wang T, Zhan X, Bu C, Lyon S, Pratt D, Hildebrand S (2015). Real-time resolution of point mutations that cause phenovariance in mice. Proc Natl Acad Sci USA.

[43] Landrum MJ, Lee JM, Benson M, Brown G, Chao C, Chitipiralla S (2016). ClinVar: public archive of interpretations of clinically relevant variants. Nucleic Acids Res.

[44] Ovilo C, Fernández A, Fernández AI, Folch JM, Varona L, Benítez R (2010). Hypothalamic expression of porcine *leptin receptor* (*LEPR*), *neuropeptide Y* (*NPY*), and *cocaine*- *and amphetamine*-*regulated transcript* (*CART*) genes is influenced by LEPR genotype. Mamm Genome.

[45] Fontanesi L, Ribani A, Scotti E, Utzeri VJ, Veličković N, Dall’Olio S (2014). Differentiation of meat from European wild boars and domestic pigs using polymorphisms in the *MC1R* and *NR6A1* genes. Meat Sci.

[46] Latorre P, Burgos C, Hidalgo J, Varona L, Carrodeguas JA, López-Buesa P (2016). Changes the enzyme kinetic and functional properties modifying fat distribution in pigs. Sci Rep..

[47] Ren J, Duan Y, Qiao R, Yao F, Zhang Z, Yang B (2011). A missense mutation in *PPARD* causes a major QTL effect on ear size in pigs. PLoS Genet.

[48] Derks MFL, Gjuvsland AB, Bosse M, Lopes MS, van Son M, Harlizius B (2019). Loss of function mutations in essential genes cause embryonic lethality in pigs. PLoS Genet.

[49] Chorev M, Joseph Bekker A, Goldberger J, Carmel L (2017). Identification of introns harboring functional sequence elements through positional conservation. Sci Rep..

[50] Ensembl gene annotation update (e!90); 2017. https://m.ensembl.org/info/genome/genebuild/2017_08_sus_scrofa_genebuild.pdf. Accessed 30 Jan 2020.

[51] NCBI Sus scrofa Annotation Release 106; 2017. https://www.ncbi.nlm.nih.gov/genome/annotation_euk/Sus_scrofa/106/. Accessed 29 Oct 2018.

[52] Hezroni H, Koppstein D, Schwartz MG, Avrutin A, Bartel DP, Ulitsky I (2015). Principles of long noncoding RNA evolution derived from direct comparison of transcriptomes in 17 species. Cell Rep..

[53] Weikard R, Demasius W, Kuehn C (2017). Mining long noncoding RNA in livestock. Anim Genet.

[54] Lopes KP, Campos-Laborie FJ, Vialle RA, Ortega JM, De Las Rivas J (2016). Evolutionary hallmarks of the human proteome: chasing the age and coregulation of protein-coding genes. BMC Genomics..

[55] Butler AB (2009). Evolution of vertebrate brains: introduction and overview. Encycl Neurosci..

[56] Guschanski K, Warnefors M, Kaessmann H (2017). The evolution of duplicate gene expression in mammalian organs. Genome Res.

