# Supplementary material for: pCADD: SNV prioritisation in Sus scrofa
Source: Genet Sel Evol. 2020 Feb 7;52:4. doi: 10.1186/s12711-020-0528-9 (PMC7006094; doi:10.1186/s12711-020-0528-9)
Supplement: Supplementary file 1 — Additional file 1. Annotation pre-processing. Description of the pre-processing procedure of the datasets used to train the pCADD model. [file 12711_2020_528_MOESM1_ESM.docx]

**pCADD: SNV prioritisation in Sus scrofa**

Christian Groß^1,2^, Martijn Derks^3^, Hendrik-Jan Megens^3^, Mirte Bosse^3^,
Martien AM Groenen^3^, Marcel Reinders^1^, Dick de Ridder^2^

^1^Delft Bioinformatics Lab, University of Technology Delft, 2600GA, Delft, The Netherlands

^2^Bioinformatics Group, Wageningen University & Research, 6708 PB, Wageningen, The Netherlands

^3^Animal Breeding and Genomics, Wageningen University & Research, Wageningen, The Netherlands

# Annotation pre-processing

To train the pCADD model, SNVs from the generated training set were annotated with features assembled from various genomic annotations. The set of putative benign SNVs (derived alleles) represent mutations that are directed back in time while simulated variants are orientated forward in time. Therefore annotations that are sensitive to these differences have to be swapped in the set of derived variants. Namely, the nucleotide reference and alternative columns (Ref, Alt ), the amino acid substitutions (nAA, oAA) and the variant effect consequence predictions made by the ENSEMBL Variant Effect Predictor v91.3 for the labels STOP Gained and STOP Lost).

Not all SNVs were be able to be annotated with all genomic annotations, therefore missing values were imputed either by fixed values (such as 0.5, 1.0 or 0, False, UD) or by the mean of the SNVs in the simulated set. False was used for boolean values, UD (undefined) for factors. To deal with factors, all columns containing factor data were OneHotEncoded. This means factor data columns were replaced by as many columns with binary values as unique factors in these columns. In addition to the imputation, indicator columns were added to the data set which contain a 1 if a particular annotation is defined for a SNV or a 0 in the cases in which they do not. These genomic annotations for which indicator columns were created are: cDNApos, CDSpos, protPos, SIFTval, Grantham and Dst2SplType_ACCEPTOR & Dst2SplType_DONOR. The last two annotations are already OneHotEncoded data columns.

The annotations minDistTSS and minDistTSE were capped at 10000 and log transformed. The VEP consequences were summarized into 14 categories/factors (Table 2) and if there are multiple consequences per SNV, the category was chosen, following the order in Table 2.

Further, combinations of annotations were created. Namely, all possible combinations of Ref and Alt categories, generating an annotation for each possible nucleotide substitution. The same was done for nAA and oAA. Added to that, combinations of the 14 different VEP consequence summaries were formed with the following annotations: cDNApos, CDSpos, Dst2Splice, GerpS, GerpN, lPhCons_noPig, mPhCons_noPig, verPhCons_noPig, lPhyloP_noPig, mPhyloP_noPig, verPhyloP_noPig, minDistTSS, minDistTSE, cDNApos, CDSpos, protPos, relcDNApos , relCDSpos , relprotPos , dnaHelT, dnaMGW, dnaProT, dnaProT.

Before model training, all data columns were scaled by dividing each value by their column standard deviation.
