# Supplementary material for: pCADD: SNV prioritisation in Sus scrofa
Source: Genet Sel Evol. 2020 Feb 7;52:4. doi: 10.1186/s12711-020-0528-9 (PMC7006094; doi:10.1186/s12711-020-0528-9)
Supplement: Supplementary file 2 — Additional file 2: Table S1. Overview of genomic annotations that build the basis for features used to train the pCADD model. Overview and short description of genomic annotations and their imputed values in the case of missing data. Table S2. Overview of the pig populations used in this study. List of pigs for which the high-frequency SNVs were added to the set of the putative benign (derived) variants to generate the training set. SNVs were called based on whole-genome sequence data. Table S3. VEP consequences summaries. VEP variant consequences, summarized into 14 categories. If multiple annotations exist for the same variant, the predicted variant consequence is selected according to the displayed hierarchy, starting at 1 and ending at 14. Table S4. Conservation score coverage of the pig genome. Coverage of the pig genome by the different conservation scores used in the pCADD model (see Table S1). Y-chromosome, mitochondrial and unplaced scaffolds were excluded in pCADD and the conservation score calculations. [file 12711_2020_528_MOESM2_ESM.docx]

**pCADD: SNV prioritisation in Sus scrofa**

Christian Groß^1,2^, Martijn Derks^3^, Hendrik-Jan Megens^3^, Mirte Bosse^3^,
Martien AM Groenen^3^, Marcel Reinders^1^, Dick de Ridder^2^

^1^Delft Bioinformatics Lab, University of Technology Delft, 2600GA, Delft, The Netherlands

^2^Bioinformatics Group, Wageningen University & Research, 6708 PB, Wageningen, The Netherlands

^3^Animal Breeding and Genomics, Wageningen University & Research, Wageningen, The Netherlands

# Additional Tables

Table S1: Overview of genomic annotations which build the basis for features used to train the pCADD model. Overview and short description of genomic annotations and their imputed values in the case of missing data.

| Annotation label | Data type | Imputed value | Annotation description |
| --- | --- | --- | --- |
| Ref | factor |  | Reference allele |
| Alt | factor |  | Observed allele |
| isTv | bool | 0.5 | Is transversion? |
| Consequence | factor |  | VEP Consequence summaries |
| GC | num | 0.414 | Percent GC in a window of +/- 75bp |
| CpG | num | 0.023 | Percent CpG in a window of +/- 75bp |
| motifECount | int | 0.0 | Total number of overlapping motifs |
| motifEHIPos | bool | False | Is the position considered highly informative for an overlapping motif by VEP |
| motifEScoreChng | num | 0.0 | VEP score change for the overlapping motif site |
| Domain | factor | UD | Domain annotation inferred from VEP annotation (ncoils, tmhmm, sigp, lcompl, ndomain = "other named domain") |
| Dst2Splice | int | 0.0 | Distance to splice site in 20bp; positive: exonic, negative: intronic |
| Dst2SplType | factor | UD | Closest splice site is ACCEPTOR or DONOR |
| oAA | factor | UD | Amino acid of observed variant |
| nAA | factor | UD | Reference amino acid |
| Grantham | int | 0.0 | Grantham score: oAA,nAA |
| SIFTcat | factor | UD | SIFT category of change |
| SIFTval | num | 0.0 | SIFT score |
| cDNApos | int | 0.0 | Base position from transcription start |
| relcDNApos | num | 0.0 | Relative position in transcript |
| CDSpos | int | 0.0 | Base position from coding start |
| relCDSpos | num | 0.0 | Relative position in coding sequence |
| protPos | int | 0.0 | Amino acid position from coding start |
| relProtPos | num | 0.0 | Relative position in protein codon |
| dnaRoll | num | 0.255 | Predicted local DNA structure effect on dnaRoll |
| dnaProT | num | 0.518 | Predicted local DNA structure effect on dnaProT |
| dnaMGW | num | 0.0365 | Predicted local DNA structure effect on dnaMGW |
| dnaHelT | num | -0.102 | Predicted local DNA structure effect on dnaHelT |
| GerpS | num | -0.805 | Rejected Substitution' score defined by GERP++ |
| GerpN | num | 1.384 | Neutral evolution score defined by GERP++ |
| GerpRS | num | 0.0 | Gerp element score |
| GerpRSpval | num | 1.0 | Gerp element p-Value |
| lPhCons_noPig | num | 0.143 | 6-taxa-Laurasiatheria PhastCons score (excl. pig) |
| mPhCons_noPig | num | 0.135 | 25-taxa-Mammalian PhastCons score (excl. pig) |
| verPhCons_noPig | num | 0.126 | 100-taxa-Vertebrate PhastCons score (excl. pig) |
| lPhyloP_noPig | num | 0.078 | 6-taxa-Laurasiatheria PhyloP score (excl. pig) |
| mPhyloP_noPig | num | 0.106 | 25-taxa-Mammalian PhyloP score (excl. pig) |
| verPhyloP_noPig | num | 0.294 | 100-taxa-Vertebrate PhyloP score (excl. pig) |
| minDistTSS | int | 10000000 | Distance to closest Transcribed Sequence Start (TSS) |
| minDistTSE | int | 10000000 | Distance to closest Transcribed Sequence End (TSE) |

Table S2: Overview of the pig populations used in this study. List of pigs whose high frequency SNPs were added to the set of the putative benign (derived) variants to generate the training set. SNPs were called based on whole genome sequence data.

| Number of individuals | Race/Breed |
| --- | --- |
| 2 | Angler Sattelschwein |
| 2 | Berkshire |
| 2 | British Saddleback |
| 2 | Bunte Bentheimer |
| 1 | Calabrese |
| 7 | Cassertana |
| 2 | Chato Murciano |
| 8 | Chinese Wild boar |
| 2 | Cinta Senese |
| 53 | Duroc |
| 2 | Gloucester Old Spot |
| 10 | Hampshire |
| 11 | Japanese Wild boar |
| 3 | Jiangquhai |
| 2 | Jinhua |
| 43 | Landrace |
| 2 | Large Black |
| 97 | Large White |
| 2 | Leping_spotted |
| 2 | Linderodsvinn |
| 7 | Mangalica |
| 10 | Meishan |
| 2 | Middle White |
| 3 | Negro Iberico |
| 1 | Nera Siciliana |
| 13 | Pietrain |
| 3 | Retinto |
| 38 | Synthetic |
| 2 | Tamworth |
| 2 | Thai domesticated pig |
| 2 | Thai Wild boar |
| 2 | Wannan spotted |
| 37 | European Wild boar |
| 2 | Xiang pig |
| 1 | Zang pig |
| 4 | NA |

Table S3: VEP consequences summaries. VEP consequences summarized to 14 categories. If multiple annotations exist for the same variant, the consequence is selected according to the displayed hierarchy, starting at 1 and ending at 14.

| Hierarchy | Abbreviation | VEP Consequence Summary |
| --- | --- | --- |
| 1 | SG | Stop Gained |
| 2 | CS | Canonical Splice |
| 3 | NS | Non-Synonymous |
| 4 | SN | Synonymous |
| 5 | SL | STOP Lost |
| 6 | S | Splice Site |
| 7 | U5 | 5’-UTR |
| 8 | U3 | 3’-UTR |
| 9 | IG | Intergenic |
| 10 | NC | Noncoding-change |
| 11 | I | Intronic |
| 12 | UP | Upstream |
| 13 | DN | Downstream |
| 14 | O | Unknown |

Table S4: Conservation score coverage of the pig genome. Coverage of the pig genome for the conservation scores used in the pCADD model (Supplementary Table 1). Y-chromosome, mitochondrial and unplaced scaffolds were excluded in pCADD and the conservation score calculations.

| Conservation score | Nr. of positions | Fraction of the total genome |
| --- | --- | --- |
| 6-taxa-Laurasiatheria PhyloP score (excl. pig) | 1,777,718,741 | 0.71 |
| 25-taxa-Mammalian PhyloP score (excl. pig) | 1,978,673,774 | 0.79 |
| 100-taxa-Vertebrate PhyloP score (excl. pig) | 1,367,857,535 | 0.55 |
| GERP | 1,043,440,638 | 0.42 |
| 6-taxa-Laurasiatheria PhastCons score (excl. pig) | 1,777,718,741 | 0.71 |
| 25-taxa-Mammalian PhastCons score (excl. pig) | 1,978,669,505 | 0.79 |
| 100-taxa-Vertebrate PhastCons score (excl. pig) | 1,390,499,379 | 0.56 |
| Golden Path Sscrofa11.1 | 2,501,912,388 |  |
