# Supplementary material for: pCADD: SNV prioritisation in Sus scrofa
Source: Genet Sel Evol. 2020 Feb 7;52:4. doi: 10.1186/s12711-020-0528-9 (PMC7006094; doi:10.1186/s12711-020-0528-9)

**pCADD: SNV prioritisation in Sus scrofa**

Christian Groß^1,2^, Martijn Derks^3^, Hendrik-Jan Megens^3^, Mirte Bosse^3^,
Martien AM Groenen^3^, Marcel Reinders^1^, Dick de Ridder^2^

^^          

^^          

^^          

# Additional Figures

FigureS 1: Prediction performances of six conservation scores on test sets, representing different regions of the genome for which different number of features are available. I: Whole test set; II: Intergenic SNVs; III: Transcribed SNVs; IV: SNVs in intron, 5’ & 3’ UTRs; V: Coding SNVs; VI: SNVs causing synonymous mutations; VII SNVs causing missense mutations.


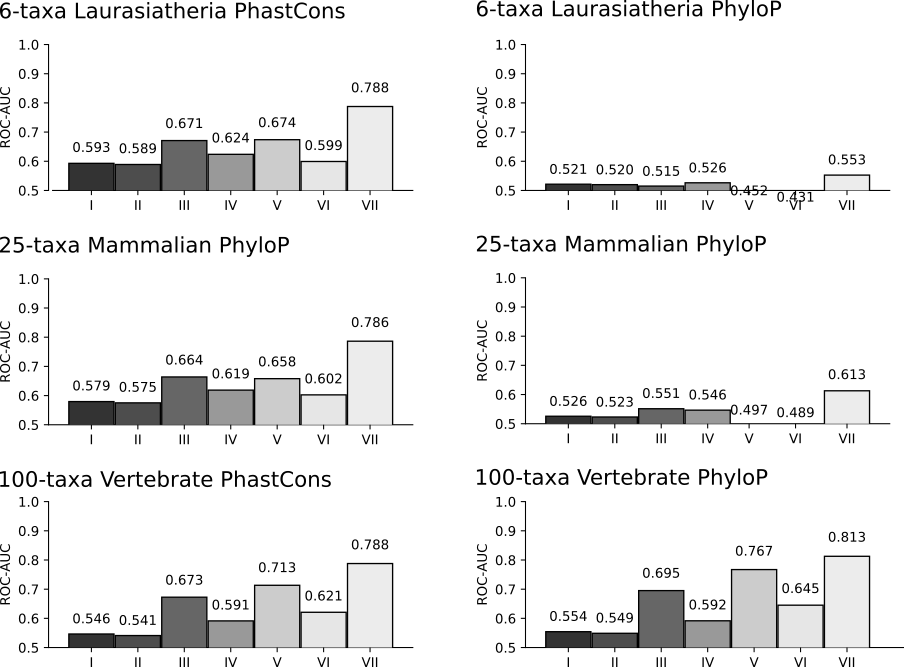


Figure S2: Codon redundancy displayed in the JBrowser genome browser using pCADD scores.The third position in a codon is more redundant than either of the other two positions. This is reflected in the scores, here an example of the end of the 2^nd^ exon of MACC1. MACC1 is located on the reverse strand.


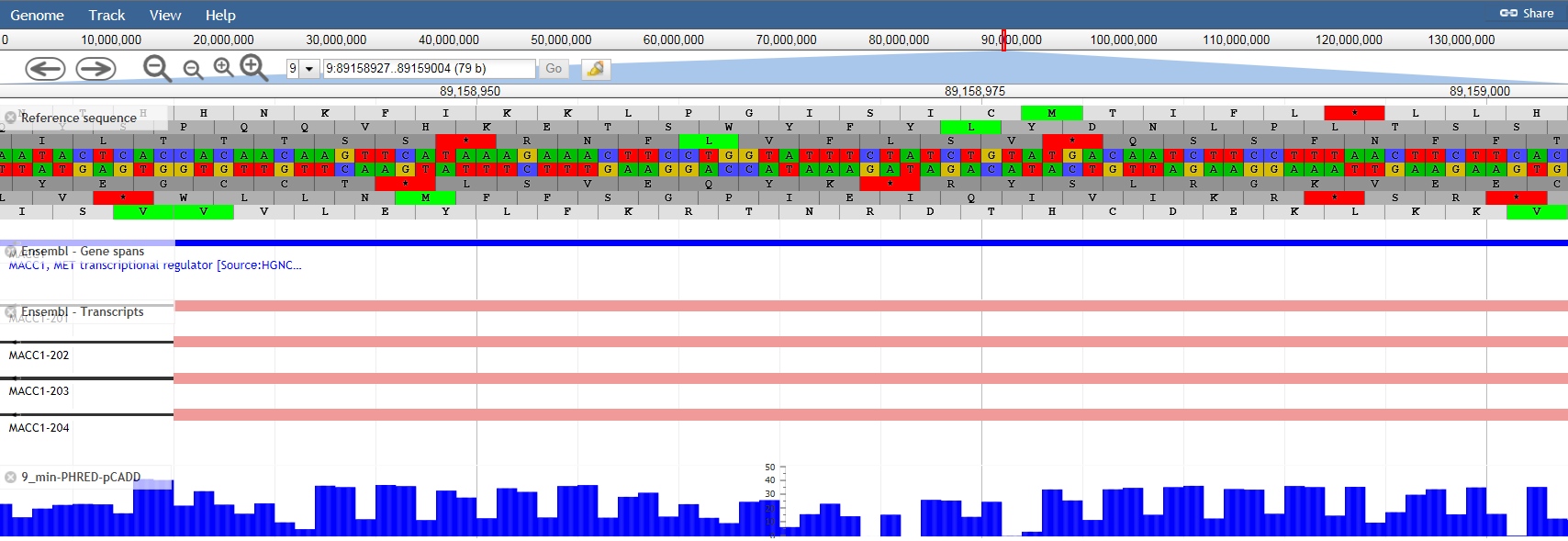


Figure S3: Effect sizes measured as ROC-AUC between the difference of pCADD scores of the three codon sites for all transcripts. The pCADD scores for the third and second codon positions differ generally the most (mean of ~0.232), thus their effect sizes have the largest absolute distance to 0.5. A ROC-AUC of 0.5 would indicate that no set of scores is larger than the other. The score indicates that the third position has a generally lower pCADD scores than the second position. The effect sizes of pCADD scores between the third and first codon positions (mean ROC-AUC ~0.277) also indicate that the third position is generally evaluated to be less deleterious than the first. In contrast, effect sizes between the second and first codon position are on average larger than 0.5 (mean of ~0.554) with the second codon position having a generally higher pCADD score than the first, which confirms that the second codon position is the most consequential when mutated. The effect sizes between the third and second codon positions as well as the third and first codon positions are more dispersed than between the second and first codon positions, probably due to the relatively larger variance in impact of a change at the third position than at the other two positions.


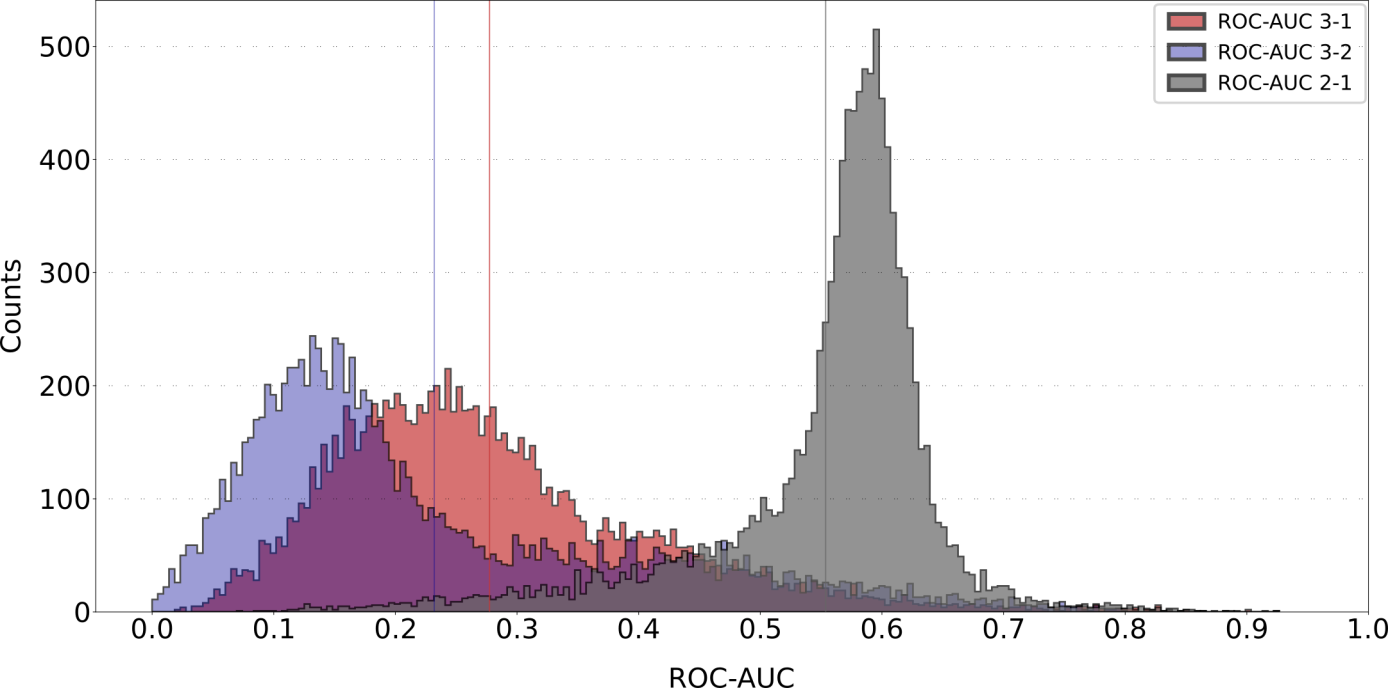


Figure S4: Histogram of conservation score distribution of (pre-)miRNA transcripts and their surrounding up- and downstream regions. Vertical lines indicate the mean values of each distribution with a mean of 0.382 for miRNA and 0.211 for Up&Down. The one-tailed Mann-Whitney U-test between both distributions returned a *p*-value of 1e-225 and a CLES of 59.54%. The conservation score used to annotate the transcripts and their surrounding regions are the 25-taxa-Mammalian PhyloP score shown in Supplementary Table 4.


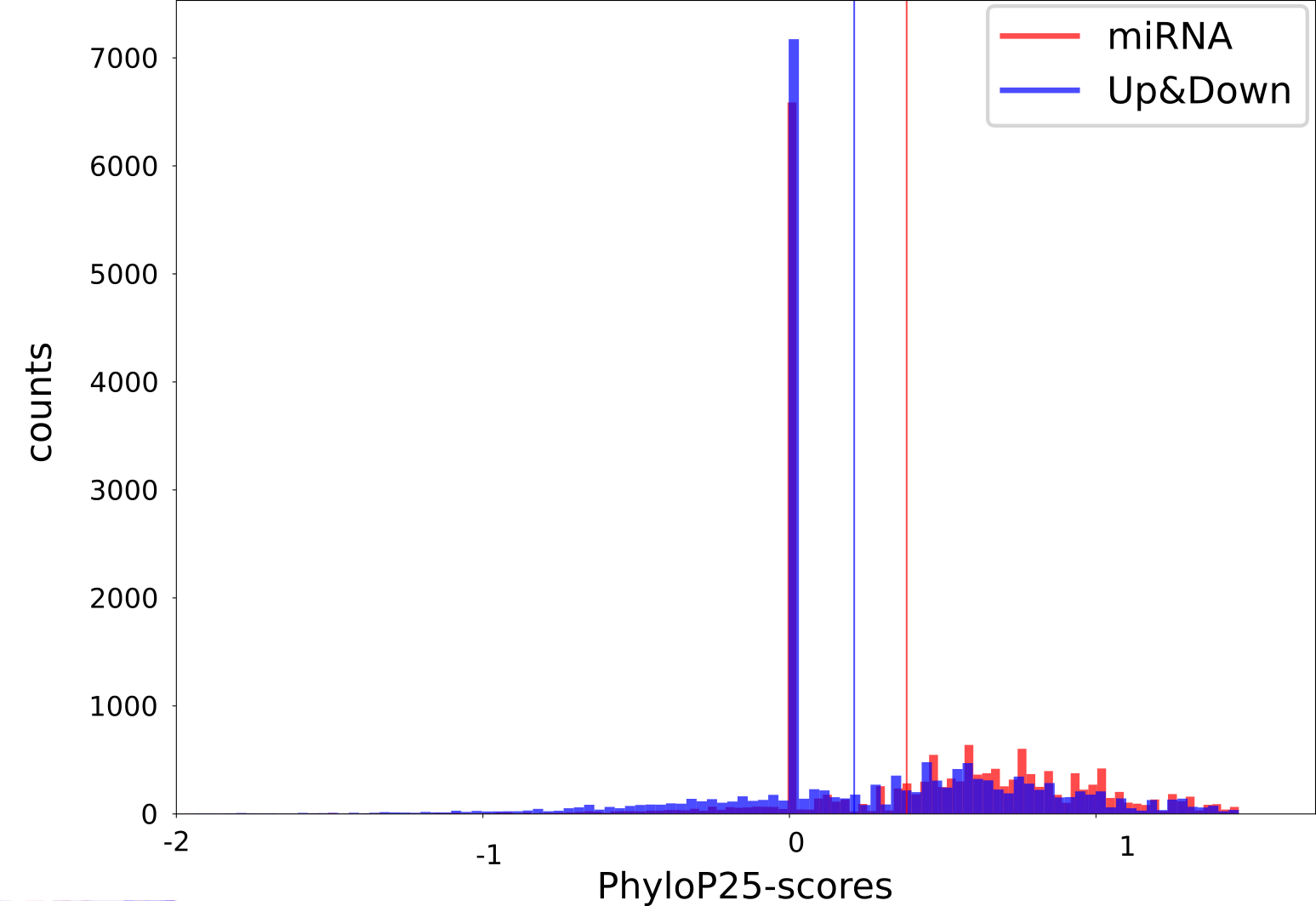


Figure S5: Comparison of the 25-taxa-Mammalian PhyloP scores per intron compared to all other introns, for the first 20 introns. The blue bar indicates the number of introns tested against the intron of interest, the red bar how many of these tests resulted in an adjusted p-value < 0.05 (scale on the left axis). As the intron position increases, the number of tests that can be conducted decreases (with the number of transcripts that have at least that many introns). In black, the normalised number of significantly enriched introns, normalized by the number of conducted tests per intron position (scale on the right axis).


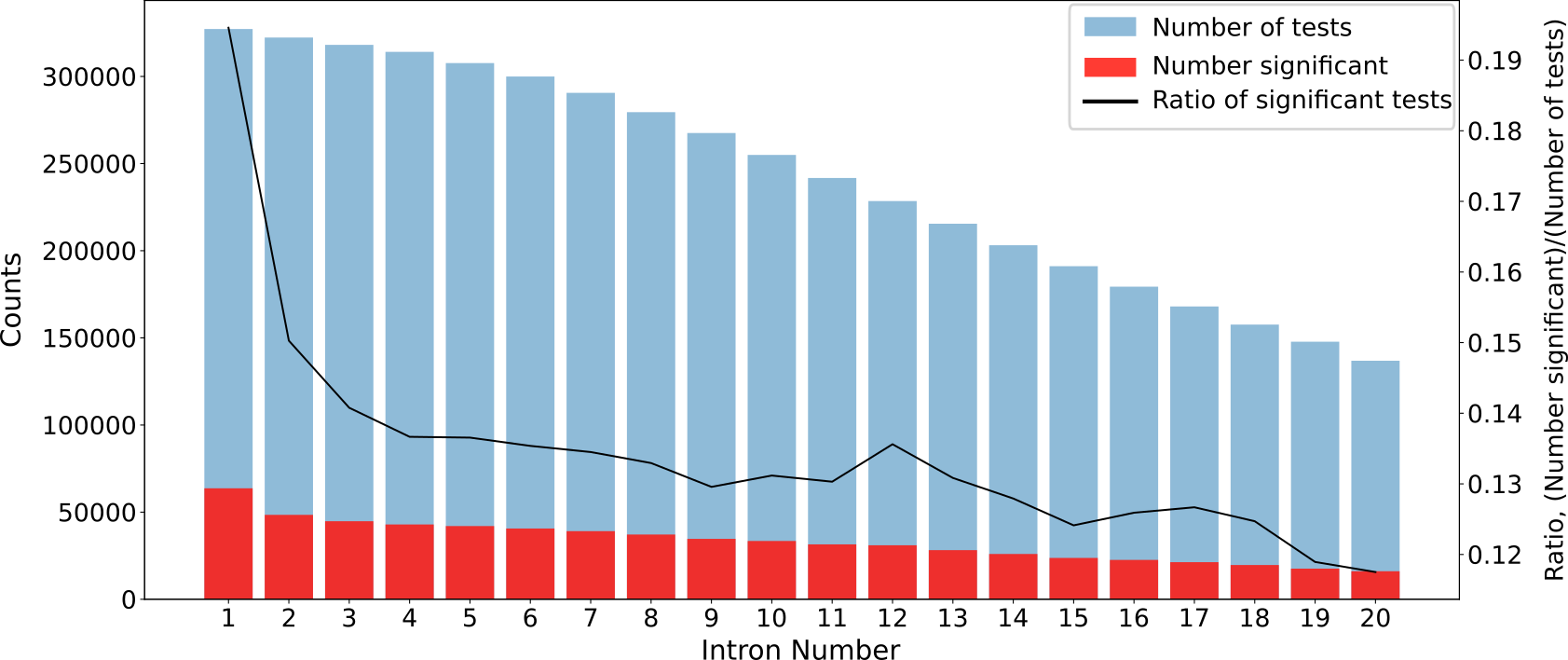


Figure S6: pCADD scores show a pattern of high scores in a presumably intergenic region. The yellow bar is indicating the location of the SNV 6:149549021T>C. It is embedded in a presumably intergenic region without any gene annotations in the pig genebuild of Ensembl and NCBI and the Ensembl genebuild of human when mapped to the human genome. The region is spiked with islands of high pPHRED scores, untypical for intergenic regions, and starts with an active enhancer region (peaks in H3K27Ac, data not part of this manuscript). The region 5’ of the enhancer site is displaying patterns as expected for intergenic regions.


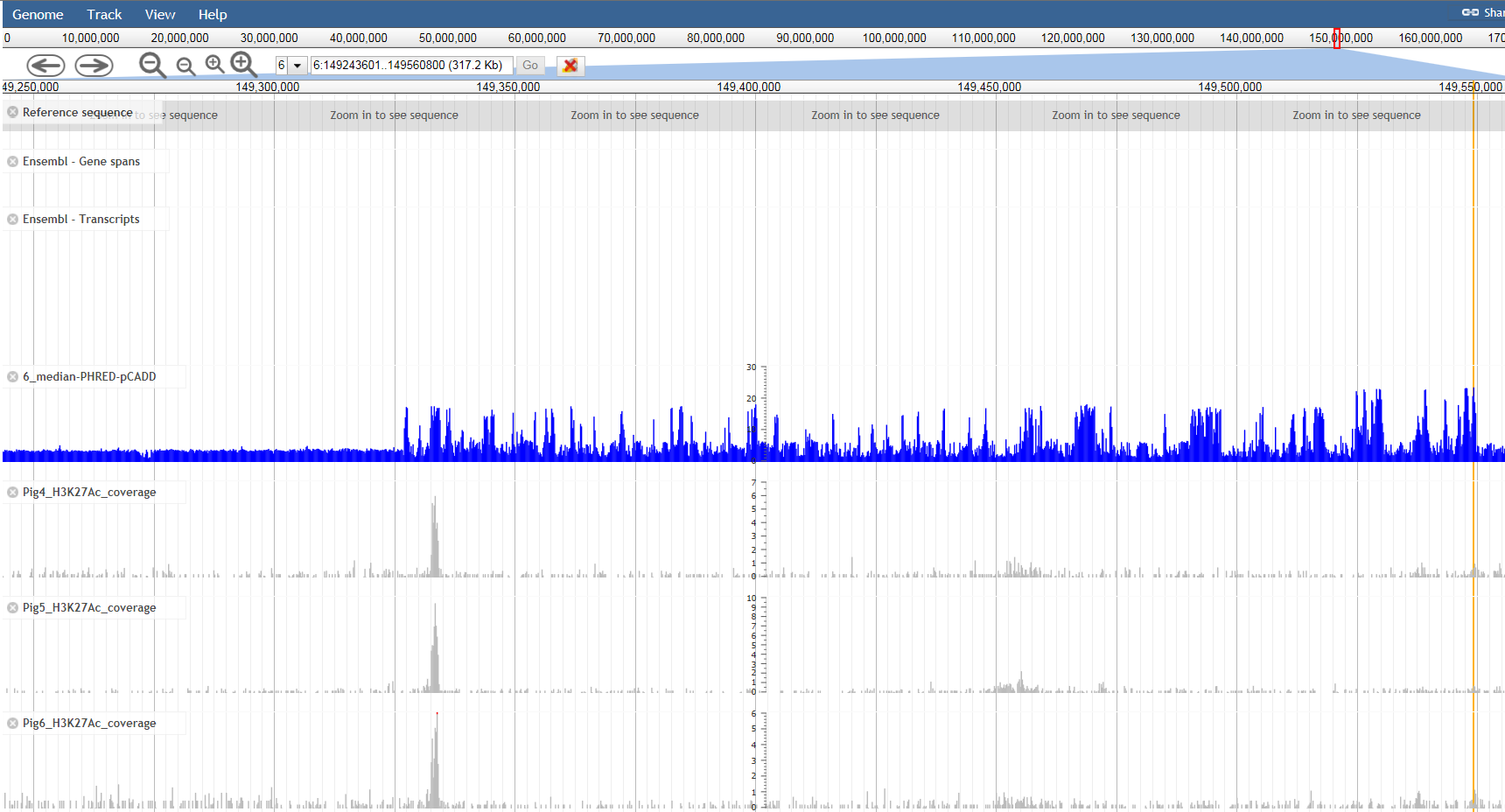

Supplement: Supplementary file 3 — Additional file 3: Figure S1. Prediction performances of six conservation scores on test sets, representing different regions of the genome for which different numbers of features are available. I: whole test set; II: Intergenic SNVs; III: transcribed SNVs; IV: SNVs in introns, 5′ and 3′ UTRs; V: coding SNVs; VI: SNVs causing synonymous mutations; VII SNVs causing missense mutations. Figure S2. Codon redundancy displayed in the JBrowser genome browser using pCADD scores. The third position in a codon is more redundant than either of the two other positions. This is reflected in the scores, here an example of the end of the second exon of the MACC1 gene. MACC1 is located on the reverse strand. Figure S3. Effect sizes measured as ROC-AUC of the pairwise comparisons of pCADD scores of the three codon sites for all transcripts. The pCADD scores for the third and second codon positions differ generally the most (mean of ~ 0.232), thus their effect sizes have the largest absolute distance to 0.5. A ROC-AUC of 0.5 would indicate that no set of scores is larger than the other. The score indicates that the third position has a generally lower pCADD scores than the second position. The effect sizes of pCADD scores between the third and first codon positions (mean ROC-AUC ~ 0.277) also indicate that the third position is generally evaluated to be less deleterious than the first. In contrast, effect sizes between the second and first codon position are on average larger than 0.5 (mean of ~ 0.554) with the second codon position having a generally higher pCADD score than the first, which confirms that the second codon position is the most consequential when mutated. The effect sizes between the third and second codon positions as well as the third and first codon positions are more dispersed than between the second and first codon positions, probably due to the relatively larger variance in impact of a change at the third position than at the other two positions. Figure S4. Histo [file 12711_2020_528_MOESM3_ESM.docx]
